# Identification and characterization of circular RNAs in association with the feed efficiency in Hu lambs

**DOI:** 10.1186/s12864-022-08517-5

**Published:** 2022-04-10

**Authors:** Deyin Zhang, Xiaoxue Zhang, Fadi Li, Xiaolong Li, Yuan Zhao, Yukun Zhang, Liming Zhao, Dan Xu, Jianghui Wang, Xiaobin Yang, Panpan Cui, Weimin Wang

**Affiliations:** 1 State Key Laboratory of Grassland Agro-Ecosystems, Key Laboratory of Grassland Livestock Industry Innovation, Ministry of Agriculture and Rural Affairs, Engineering Research Center of Grassland Industry, Ministry of Education, College of Pastoral Agriculture Science and Technology, Lanzhou University, Lanzhou, 730020 China; 2 College of Animal Science and Technology, Gansu Agricultural University, Lanzhou, 730070 Gansu China; 3 Engineering Laboratory of Sheep Breeding and Reproduction Biotechnology in Gansu Province, Minqin, 733300 China

**Keywords:** Hu sheep, circRNA, Residual feed intakes, Liver, *RTP4*

## Abstract

**Background:**

Circular RNA (circRNA), as a new members of noncoding RNA family, have vital functions in many biological processes by as microRNA sponges or competing endogenous RNAs (ceRNAs). However, little has been reported about the genetic mechanism of circRNAs regulation of feed efficiency in sheep.

**Results:**

This study aimed to explore the expression of circRNAs in the liver of Hu sheep with High-RFI (High residual feed intake) and Low-RFI (Low residual feed intake) using transcriptome sequencing. A total of 20,729 circRNAs were identified in two groups, in which 219 circRNAs were found as significantly differentially expressed. Several circRNAs were validated by using RT-PCR, sanger sequencing and RT-qPCR methods. These results demonstrated that the RNA-seq result and expression level of circRNAs identified are reliable. Subsequently, GO and KEGG enrichment analysis of the parental genes of the differentially expressed (DE) circRNAs were mainly involved in immunity response and metabolic process. Finally, the ceRNA regulatory networks analysis showed that the target binding sites for miRNA such as novel_41, novel_115, novel_171 and oar-miR-485-3p in the identified DE cirRNAs. Importantly, two metabolic (*SHISA3* and *PL*EKHH2) and four (*RTP4*, *CD274*, *OAS1*, and *RFC3*) immune-related target mRNAs were identified from 4 miRNAs. Association analysis showed that the polymorphism (*RTP4* c.399 A > G) in the target gene *RTP4* were significantly associated with RFI (*P* < 0.05).

**Conclusions:**

Analysis of sequencing data showed some candidate ceRNAs that may play key roles in the feed efficiency in sheep by regulating animal immune and metabolic. These results provide the basis data for further study of the biological functions of circRNAs in regulating sheep feed efficiency.

**Supplementary Information:**

The online version contains supplementary material available at 10.1186/s12864-022-08517-5.

## Background

The cost of feed is a major expend in the indoor sheep production industry, accounting for about 65–70% of total expenditures [1]. Due to this cost, the selective breeding of sheep with high feed efficiency has become an important objective of breeding programs. Feed conversion rate (FCR) and residual feed intake (RFI) are two the most commonly used phenotypes for quantifying feed efficiency in livestock species [2]. FCR is the ratio between the total of feed intake and the individual weight gain over a specific time, RFI index was first proposed by Koch et al. (1963) and has successfully applied to the artificial selection of feed efficiency in livestock, which is defined as the difference between actual feed intake and predicted feed intake of each individual requirement for maintaining growth and production [3, 4]. The RFI is an accurate and sensitive index compared with the FCR, because the FCR’s strong negative correlation with body weight gain, and is a ratio traits, it has certain limitations on genetic selection for evaluating FE [5, 6], while the RFI is a trait independent of other production traits. Thus, RFI has gained popularity in recent years, particularly among geneticists, and has been considered as a desirable criterion for the genetic improvement of feed efficiency in livestock breeding.

RFI is a complex quantitative trait, influenced by many biological factors [7]. The digestibility and metabolism processes as well as animal health status are important factor affecting RFI. The liver plays an important role in the metabolic and animal innate immunity, which have vital effects on sheep digestion and metabolism of nutrients. RNA sequencing (RNA-seq) is acknowledged as a mature technology and widely applied to understand the regulatory molecular mechanisms underlying of feed efficiency, and the genetic and breeding approaches are effective to improve feed efficiency. Many studies on the genetic variations affecting feed efficiency of livestock at the mRNA level [8, 9]. CircRNA was first discovered in plant viruses [10], and plays an important role in the regulate expression genes associated with several biological processes as a special of noncoding RNA. To date, many literature showed that circRNAs are mainly related to biological processes such as reproduction [11, 12], growth development [13], milk production [14] and wool follicle development [15] in sheep. However, it is not known about how circRNA regulates feed efficiency in sheep.

Hence, to explore the underlying genetic mechanisms of regulation of feed efficiency in sheep. In the present study, RNA-seq was performed to study the circRNAs of liver samples from Low-RFI and High-RFI lambs. We also describe the genomic features chromosome and length distribution of circRNAs in the liver tissue in sheep. Simultaneously, reverse transcriptase PCR amplification and DNA sequencing technologies were used to validate the presence of circular RNA. Subsequent bioinformatics analyses were performed of the parent genes of the differentially expressed circRNAs, and further constructed a circRNA-miRNA-mRNA interaction network. These findings help us better understand the genetic mechanisms that regulate RFI at the circRNA levels.

## Results

### Characterization of circRNAs in the *ovine* liver tissue

**Fig. 1:**
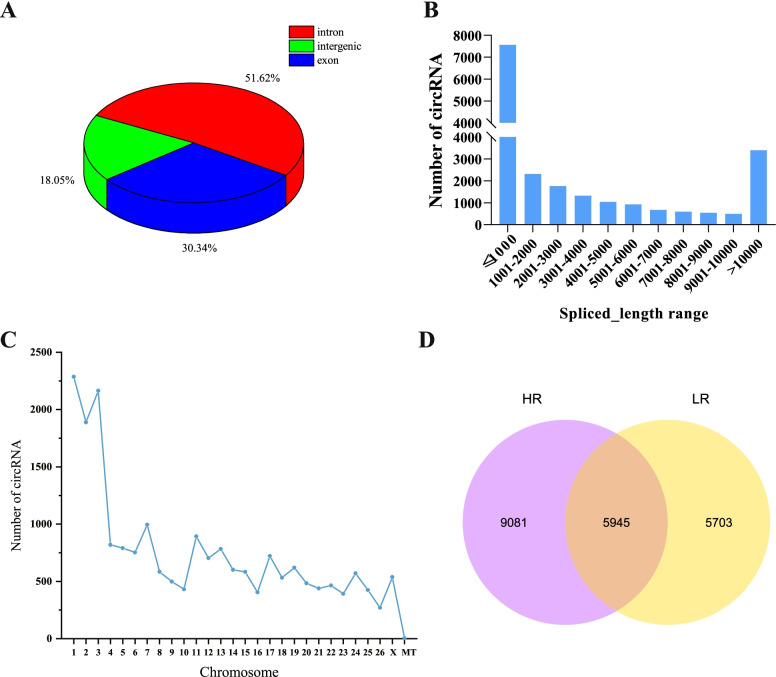
Characteristics of circRNAs in the sheep in the ovine liver tissue with High-RFI and Low-RFI. **A** The proportion of introns, exons and intergenic of circRNAs. **B** The number of circRNAs within different spliced-length ranges. **C** Chromosomal distribution of circRNAs. **D** The Venn diagram of two comparisons and the number of annotated circRNAs

To understand the circRNA expression patterns of liver tissue from the male Hu lambs with different residual feed intakes. We carried out RNA-seq on liver samples from three Low-RFI and three High-RFI lambs. A total of 243 million raw reads were obtained from RNA-seq data. Approximately 227 million clean data obtained by removing adapter and low quality sequences were mapped to the *ovine* reference genome (*Oar_v4.0*). Table 1 summarizes the total number of reads generated from six samples, and each sample yielded more than 30 million raw reads data. The average GC content was 62.72% (Table 1). After removing the linear RNA and ribosomal RNA, we detected a total of 20,729 circRNAs from the high quality of the RNA-seq data by find_circ, and the majority of circRNAs consisted mainly of introns and exons, whereas a small fraction are composed of intergenic sequences (Fig. 1A). The length of most candidate circRNAs were mainly focused below 1000 nt (Fig. 1B). The circRNAs in the present study were extensively distributed on 26 autosomes and sex chromosomes, of which most circRNAs were concentrated on chromosomes 1 to 3 (Fig. 1C).

**Table 1:** Summary statistics of the RNA-Seq data

Sample	Raw Reads	Clean Reads	Error rate (%)	Q20 (%)	Q30 (%)	GC content (%)
LR1	38,414,647	36,495,858	0.02	98.34	94.99	62.51
LR2	32,883,576	30,287,731	0.02	98.36	95.03	62.82
LR3	47,310,528	43,710,001	0.02	98.33	94.98	62.85
HR1	41,475,895	38,357,412	0.02	98.27	94.84	63.4
HR2	40,148,898	37,688,849	0.02	98.26	94.86	62.46
HR3	43,391,501	40,465,965	0.02	98.35	95.09	62.28
Total	243,625,045	227,005,816				62.72

### Differentially expressed analysis of circRNAs

**Fig. 2:**
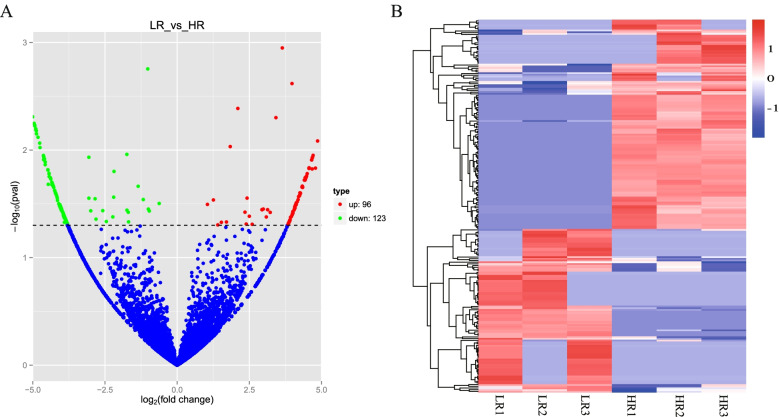
Differentially expressed gene analysis in the ovine liver tissue with High-RFI and Low-RFI. **A** Volcano plot of circRNAs differential expression results. **B** Clustering heat-map of the DE circRNAs

From the six liver samples, we obtained 15,026 and 11,648 circRNAs from the Low-RFI and High-RFI groups, respectively. Of all circRNAs, 5945 circRNAs were co-expressed in the liver of both the Low-RFI and High-RFI lambs (Fig. 1D), 219 circRNAs were significantly differentially expressed (|log_2_Fold Change|≥ 1 and *p*-value < 0.05) when comparing Low-RFI and High-RFI groups. Compared to High-RFI group, the expression levels of 96 circRNAs were up-regulated in Low-RFI group, while 123 circRNAs were down-regulated (Fig. 2A). The hierarchical clustering heatmap analysis suggested that the expression patterns of the differentially expressed circRNAs were clearly differentiated and aggregated between Low-RFI and High-RFI groups (Fig. 2B).

### Validation of differentially expressed circRNAs

**Fig. 3:**
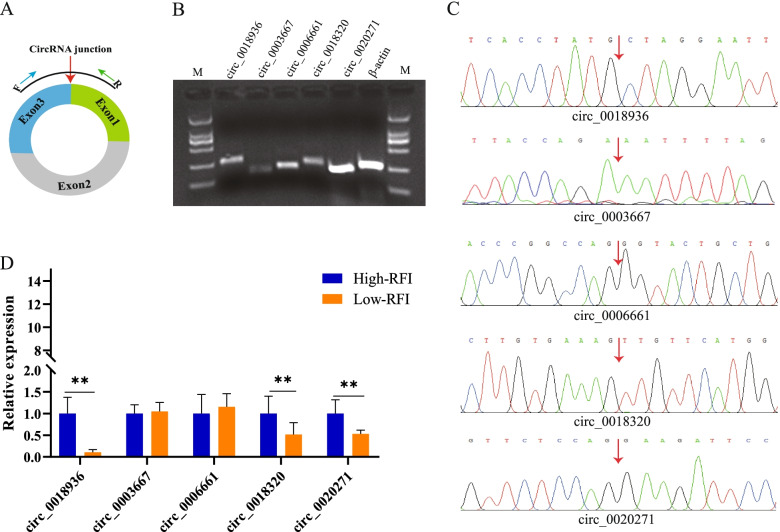
Verification of circRNAs data from transcriptome sequencing. **A** Divergent primers used to amplify the circular junctions. **B** Visualization of RT-PCR amplification of circRNAs on a 1.5% agarose electrophoresis gel. **C** Head-to-tail junctions were confirmed by Sanger sequencing. **D** The expression levels of DE circRNAs in liver tissues of groups with different residual feed intakes. Values are means ± SE. * *P* < 0.05, ** *P* < 0.01

To validate the presence of circRNAs circular structures (Fig. 3A), we randomly selected five DE circRNAs to perform RT-PCR amplification and DNA sequencing. The 1.5% agarose gel electrophoresis results showed all five circRNAs can be checked and have a single band of the expected size (Fig. 3B). Simultaneously, the RT-PCR amplification product was sequenced using Sanger sequencing, the sequencing results was consistent with the sequences from RNA-Seq, which confirmed the presence of head-to-tail back-splicing of these circRNAs (Fig. 3C). Finally, the five circRNAs were subjected to quantitative RT-PCR (real-time quantitative PCR) analysis using the same primer as RT-PCR amplification, the results from the RT-qPCR for five circRNAs was consistent with the trends obtained from the RNA-seq analysis (Fig. 3D). These data demonstrated that the RNA-seq result and expression levels of candidate circRNAs are reliable in the present study.

### Enrichment analysis of for source genes of differentially expressed circRNAs

**Fig. 4:**
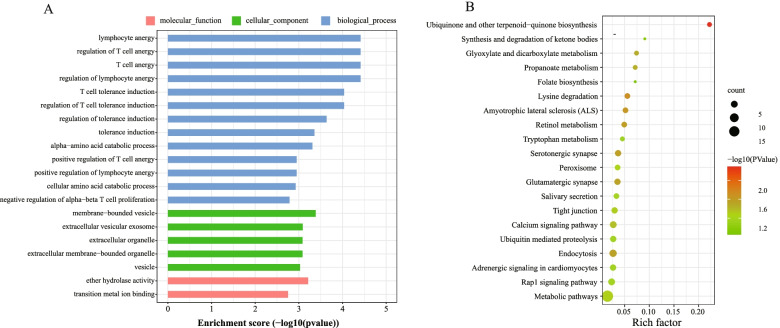
Function enrichment analysis for the source genes of circRNAs. **A** Gene ontology (GO) distribution of source genes. **B** Kyoto Encyclopedia of Genes and Genomes (KEGG) classification of source genes

To further investigate biological functional of host genes of the differentially expressed circRNAs in the Low-RFI group and the High-RFI group, we performed GO and KEGG pathways enrichment analysis for 219 DE circRNAs hosting genes. The GO analyses showed a total of 233 terms, including 133 about biological process (BP), 25 about cellular component (CC), and 75 GO about molecular function (MF) were significantly enriched (*P* < 0.05), Fig. 4A shows the top 20 most enriched categories that were related to immunity response, such as the lymphocyte anergy, regulation of T cell anergy as well as T cell energy and so on. KEGG pathway assessment indicated that the host genes for DE circRNAs were significantly enriched in ubiquinone and other terpenoid-quinone biosynthesis, lysine degradation, retinol metabolism, glyoxylate and dicarboxylate metabolism, propanoate metabolism and other signaling pathways (Fig. 4B), and some derived genes were engaged in the metabolic pathway, but not significantly, which suggest that some circRNAs are involved in the process of energy metabolic, and then play a regulatory role.

### Analysis of circRNA-miRNA-mRNA networks

**Fig. 5:**
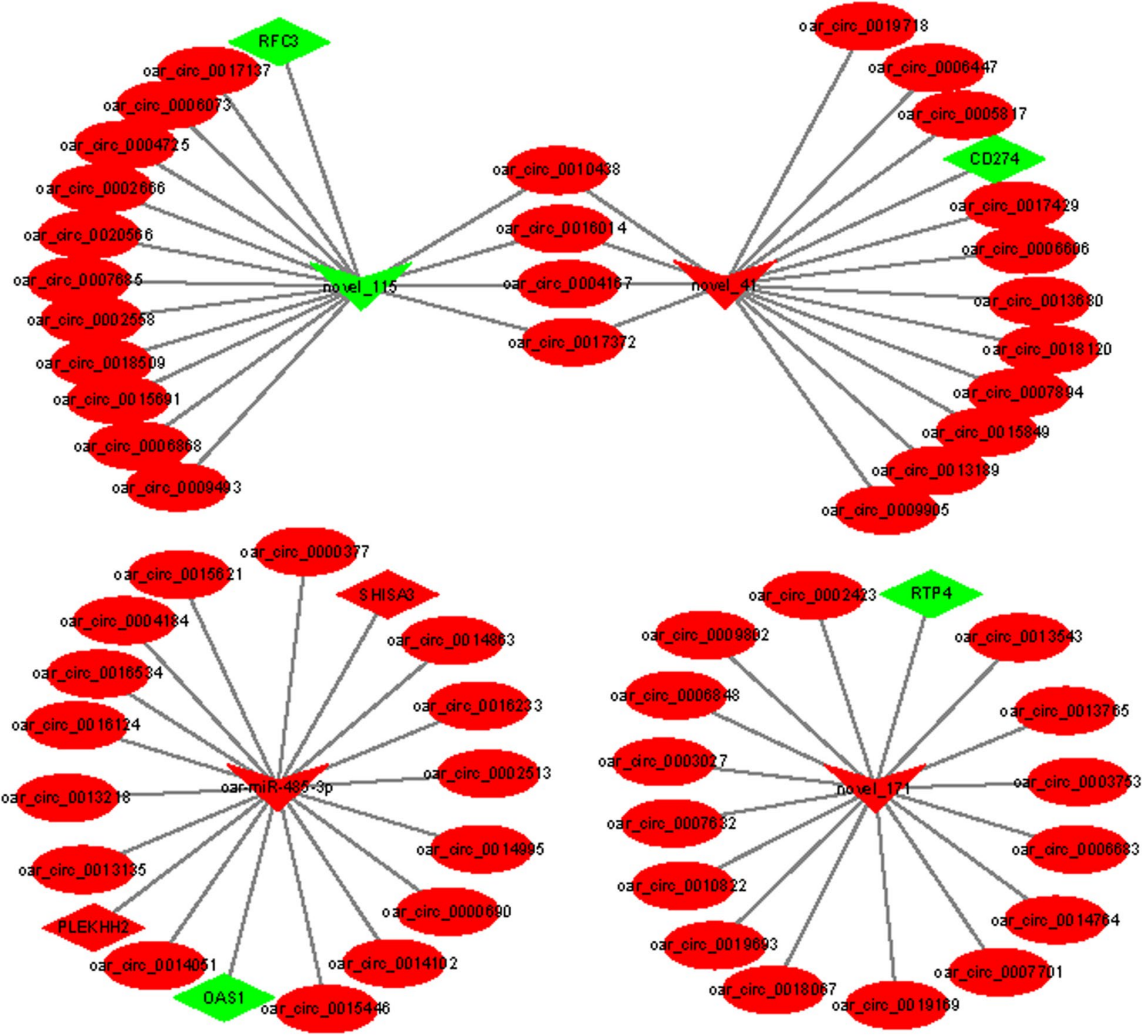
The interaction network of circRNA–miRNA–mRNA. Diamond represent mRNA, ellipse represent circRNAs, V represent miRNA, the green and red indicate down-regulated and up-regulated, respectively

The biological roles of DE circRNA could be obtained by exploring the function of target miRNAs and mRNAs. We constructed ceRNA regulatory networks of circRNA-miRNA-mRNA by the integration of our previous mRNA data and the unpublished miRNA data. A total of 590 interactions between 147 circRNAs and 4 DE miRNAs were identified (Supplementary Table S1). Notably, some circRNAs (circ_0010438, circ_0016014, circ_0017372 and circ_0004167) contained at least two potential binding sites for four differentially expressed miRNAs (Fig. 5), and the four miRNAs have six encoding protein genes (*SHISA3, PLEKHH2, RTP4*, *CD274*, *OAS1*, and *RFC3*), these genes play a role in metabolism process and immunity function. thereby suggesting that these circRNAs may be involved in metabolic and immunity process of liver by sponging multiple miRNAs.

### SNP identification and association analysis

**Fig. 6:**
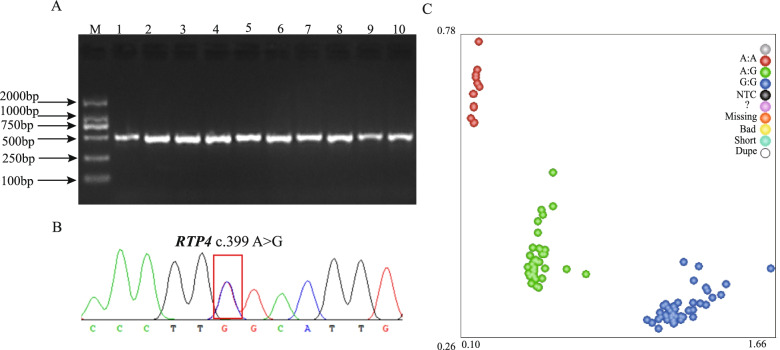
Single nucleotide polymorphism identification of the RTP4 Genes. **A** PCR amplification of mixed DNA of RTP4 gene. **B** Sequencing peak images for RTP4 gene. **C** Genotyping of RTP4 c.399 A > G SNPs using Kaspar technology

To further verify the effect of target genes of our above network results on the feed efficiency in the enlarged experimental population. The sequences of 486 bp PCR fragments were amplified from the mixed DNA from 20 Hu sheep as templates by using PCR primer pairs in Table 4 (Fig. 6A), the PCR products were sequenced by Tsingke (Xi’an, China) to scan for SNPs (single nucleotide polymorphisms) in the target genes. The sequencing results showed that a novel SNP (c.399 A > G) was identified in ovine the *RTP4* gene (Fig. 6B), and subsequently genotyped with KAspar assays in the enlarged experimental population (*n* = 1220), three genotypes were obtained: the red, green and blue dots representing AA, AG and GG genotypes, respectively (Fig. 6C). A total of 1194 individuals were genotyped successfully and used for association analysis, the result of the association analysis found that the polymorphism *RTP4* c.399 A > G was not associated with FCR (*P* > 0.05), whereas the polymorphism *RTP4* c.399 A > G was significantly associated with RFI (*P* < 0.05) (Table 2), and in this locus, sheep with the GG genotypes were significantly greater than AA genotype carriers on RFI. However, the difference between AG individuals and AA and GG individuals was not significant. This result showed that allele G contributes to higher phenotype values compared with that in the allele A.

**Table 2:** Association results between genotypes at c.399 A > G polymorphism of the ovine *RTP4* and feed efficiency traits

Gene/loci	Genotype	No	RFI	FCR
*RTP4* c.399 G > A	AA	135	-0.00962 ± 0.090^b^	5.935 ± 0.679
	AG	513	-0.00083 ± 0.089^ab^	5.919 ± 0.644
	GG	546	0.00983 ± 0.087^a^	5.988 ± 0.866

*Note*. Letters with different genotypes in same trait (a, b) means significantly at *P* < 0.05

## Discussion

As is widely known, the RFI is a vital and accurate index that is increasingly recognized as an alternate evaluate of feed efficiency of livestock [16, 17]. Considering the importance of RFI to evaluate feed efficiency on the economics of sheep production, and the estimated heritability of RFI in sheep populations is moderate, it is difficult to change this feature through traditional selection. Therefore, it is of great significance to elucidate the genetic mechanisms underlying RFI at the molecular level to improve feed efficiency.

With breakthroughs in high-throughput sequencing techniques, RNA-seq technology has been used successfully for identifying and characterizing circular RNAs [18]. In recent years, though many unique circRNAs have been identified in a variety of species based on the reference genome has been assembled, and annotated. However, there are no studies concering the role of circRNAs in the process of RFI in sheep. Herein, we identified and described the expression patterns of circRNAs in extreme RFI sheep liver tissue using RNA-seq, as well as bioinformatics analysis. A total of 219 DE circRNAs was found in the Low-RFI and High-RFI groups. The total of number of circRNAs here was less than other tissues of sheep, for example, Li et al. identified that 5086 circRNAs were differentially expressed in longissimus dorsi muscle of sheep at different development periods [19]. This result is consistent with previous studies in pigs [20] and humans [21] that have found circRNAs have specific expression patterns in differential tissues. These circRNAs in the present study were mainly distributed over 1 to 3 chromosomes, which was associated with the length of these chromosomes, and the distribution of circRNAs was consistent with what was described by Li et al. [22]. Furthermore, the RT-PCR, DNA sequencing as well as RT-qPCR were performed to confirm the particular circular structure and sequencing data of these circRNAs. We results demonstrated the presence of circRNAs circular structure and the reliability of sequencing data.

Previous studies showed that circRNAs may affect the biological functions by regulating the expression level of their host gene [23]. Functional enrichment analysis was performed for the host genes of differentially expressed circRNAs. The GO annoation results indicated the main functions for the source genes of circRNAs were involved in immune response. Existing literature showed that activating the immune system requires considerable energy [24], and the transfer of nutrients from growth to immune-related processes may increase maintenance requirements in animals during the immune response [25]. And many studies showed the immune response may be important factors contributing to the difference in feed efficiency [26, 27]. KEGG enrichment indicated that the parental genes of circRNAs were mainly related to metabolism-related pathways, which may be related to the metabolic ability of the liver, because the liver is the largest metabolic organ, and plays an important role in protein, carbohydrate, amino acid as well as fat metabolism [28, 29]. This is consistent with previous studies that have found that energy is the limiting factor that affects the feed efficiency of animals.

Finally, to further understand how circRNAs is to regulate feed efficiency. We constructed circRNA-miRNA-mRNA interaction network based on competing endogenous RNAs (ceRNAs) to reveal the main functions for these DE circRNAs. Some literatures demonstrated that another major function of circRNAs is to act as miRNA sponges to indirectly regulate expression of downstream target gene for miRNAs [30]. In the interaction network, 219 DE circRNAs targeting 4 DE miRNAs were found, interestingly, we found that some circRNAs contain two target sites of miRNAs (novel_41, novel_115, novel_171 and oar-miR-485-3p), consistent with the previous findings that some circRNAs have multiple binding sites for miRNAs [31]. Simultaneously, 6 targeted key genes (*SHISA3, PLEKHH2, RTP4*, *CD274*, *OAS1*, and *RFC3*) were identified from 4 miRNAs, and these genes were mainly related to metabolism process and immunity function. Energy metabolism and immune response are important process that affect the feed efficiency [2]. In the present study, two target genes (*SHISA3* and *PLEKHH2*) have been identified as related to metabolism process. *SHISA3* (shisa family member 3) gene encodes a single-transmembrane protein which is one of nine members of a family of transmembrane adaptors. Li et al., reported *SHISA3* gene is associated with intramuscular fat percentage of beef, suggesting the function of *SHISA3* might be related with adipose deposition [32]. *PLEKHH2* (Pleckstrin homology, MyTH4 and FERM domain containing H2) is a protein coding gene, and play a role in linking podocyte foot processes to the glomerular basement membrane. However, few studies have reported the function of *PLEKHH2. RTP4* (Receptor transporter protein 4) as an innate antiviral effector [33], it was related to the flavivirus replication, binds viral RNA, and inhibits viral genome amplification [34], and plays a role in diverse viral infections [35]. In addition, Li et al. reported that *RTP4* was associated with immune cell infiltration [36]. This suggests that *RTP4* might be involved in immunity response process, while previous study indicated that activation of the immune response results in low feed efficiency due to increased maintenance requirements [26, 37]. In addition, nutrients shifted away from growth toward the immune-related processes may reduce feed efficiency in animals during the immune response [25]. In the present study, we found that a nonsynonymous SNP c.399 A > G mutation in the *RTP4* gene was significantly associated to RFI. Therefore, we speculated circRNAs, as a miRNA sponge, by regulating the expression of downstream target gene, thus affect immune response and metabolic functions of the liver. Nonetheless, the specific mechanisms need to be studied in future via experiments.

## Conclusion

In the present study, we identified and examined the expression patterns, genomic characteristics, and length distribution of circRNAs in sheep liver. Meanwhile, Function enrichment indicated that the source genes of circRNAs were mainly related to immunity response and metabolism-related pathways. Interestingly, the polymorphism (c.399 A > G) in the target gene *RTP4* were significantly associated with RFI, which lay the foundation for the study of the regulation feed efficiency by circRNAs.

## Methods

### Data collection and samples obtained

In the present study, the animal performance and liver tissues of circular RNA sequencing were the same as those investigated in our previous study [38]. Briefly, 137 healthy male Hu lambs were raised according to the procedures described by Zhang et al. (2019), and the feed intake (FI), initial body weight (IBW) and final body weight (FBW) of all lambs were determined in the experimental period. Simultaneously, the average daily feed intake (ADFI), average daily gain (ADG), mid-test metabolic body weight (MBW), feed conversion ratio (FCR) and residual feed intake (RFI) were calculated, the formulae used was: ADFI = FI/N, ADG = (IBW—FBW)/N, MBW = [0.5*(IBW + FBW)]^0.75^, FCR = ADFI/ADG, and the RFI was calculated using a linear regression model according to the data on ADG, MBW and ADFI, the basic model used was: Y_i_ = β_0_ + β_1_MBW_i_ + β_2_ADG_i_ + e_i_, in the formulae and model above, N is the experimental period (days), Y_i_ is the ADFI of the i^th^ sheep; β_0_ is the regression intercept; β_1_ is the regression coefficient for MBW; β_2_ is the regression coefficient for ADG and e_i_ is the random error associated with the i^th^ animal. At the end of the experimental period, all lambs were slaughtered and the liver tissue were immediately frozen in liquid N_2_, and stored at − 80℃ until RNA isolation. For RNA-sequencing, two extreme groups were selected with both High-RFI (0.20 ± 0.02, *n* = 3) and Low-RFI (-0.25 ± 0.05, *n* = 3) sheep according to the RFI value from these individuals. The phenotype data of the other batches were collected after RNA sequencing to expand the validation experimental population of association analysis. Briefly, a total of 1220 male Hu sheep were randomly selected from Lanzhou Tianxin Sheep Industry Co. Ltd., Gansu Sanyangjinyuan Husbandry Co. Ltd., Shandong Runlin Sheep Industry Co. Ltd., Gansu Zhongsheng Huamei Sheep Industry Development Co. Ltd., Gansu Zhongtian Sheep Industry Co. Ltd., and Wuwei Pukang Sheep Industry Co. Ltd. To collect phenotypic data, all lambs were transferred to Minqin Defu Agriculture Co. Ltd. after weaning at 56 days old, and raised and housed indoors in individual pens (0.8 × 1 m) until 6 months old age. Throughout the experiment, all lambs have the same feeding model and management conditions according to our previously studies. At the end of the experimental period, venous blood samples were collected for extracting DNA.

### Library construction and circRNAs sequencing analysis

Total RNA from each liver tissue was isolated TRIzol (Invitrogen, Waltham, MA, USA) based on the manufacturer's procedure. The total RNA purity and quality was detected using the Agilent 2100 Bioanalyzer (Agilent, Santa Clara, CA, USA) and RNA Nano 6000 Assay Kit, respectively. For circRNA sequencing, firstly, we take approximately 5 µg high quality RNA per sample as input material and use the 15 U Rnase R (Epicentre, USA) for each RNA sample to digest linear RNA. Secondly, ribosomal RNA was removed by Epicentre Ribozero™ rRNA Removal Kit (Epicentre, USA). The remaining RNAs were used to construct a cDNA library of circRNAs, and the average fragment size for final cDNA library was 250–300 bp. Finally, the cDNA library of circRNAs were 150 bp paired-end sequenced using an Illumina HiSeq 2500 platform (Illumina San Diego, CA, USA) by Beijing Novogene Science and Technology Co., Ltd. (Beijing, China). The adaptor sequences and low quality reads with quality scores < Q20 were removed, the remaining reads were mapped to the Ovis aries reference genome (*Oar_v4.0*) using TOPHAT (v2.0.9) (tophat -o./ file1.fastq,file2.fastq Bowtie2Index/genome). Subsequently, the Find_circ software (python find_circ.py -G chomosomes.fa -p prefix -s find_circ.sites.log > find_circ.sites.bed 2 > find_circ.sites.reads) was used to identify circRNA following the steps. The 20 bp anchor sequences from both ends for each unmapped read aligns again with the *Ovis aries* reference sequence. The 5' end of the anchor sequence is aligned to the reference sequence, meanwhile, the 3' end of the anchor sequence is aligned to the upstream of this site, and there are splicing sites (GT-AG) between the breakpoints of the reference sequence, which this read is considered as candidate circRNA. Finally, the characterization of circRNAs was statistically analyzed, the expression level of each identified circRNAs were calculated using TPM method [39], the DESeq R package (https://github.com/nyudanin/RNASeq_DESeq2/blob/master/2017-11-03_DESeq2.R) was performed to identify DE circRNAs between Low-RFI and High-RFI liver tissue. The significant DE circRNAs were selected using the parameters of a |log_2_Fold Change|≥ 1 and *p*-value < 0.05.

### Validation of circRNAs presence using RT-PCR amplification and Sanger sequencing

To validate the presence of circRNAs circular structures. Total RNAs were extracted from liver tissues of sheep using TRIzol reagent (Invitrogen), one microliter of total RNA was used as templates to synthesize complementary DNA (cDNA) with a EvoM-MLV RT kit with gDNA Clean for qPCR (Accurate Biotechnology Co., Lth, Hunan, China). Five divergent primers used for circRNAs PCR were designed using Oligo 7.0 software, and were synthesized by Tsingke (Xi’an, China) and listed in Table 3. The cDNA products were amplified using circRNA PCR primers, the amplified products were checked by agarose gel (1.5%) electrophoresis and then sequenced using Sanger sequencing. The sequences data of amplified products were aligned to the RNA-seq data and the sheep reference genome with DNAMAN (v 9.0) software to determine the authenticity of the location of the junction sites in the circular RNAs.

**Table 3:** RT-PCR primers and annealing temperatures for DE circRNAs

CircRNAs	Forward 5' → 3'	Reverse 5' → 3'	product length (bp)	Tm/°C
*circ_0018936*	AGTCCCTGAGTTACGCCTT	TGGCCTTACCGATGATGACA	253	55
*circ_0003667*	TCCTTCTCTGGGGTTCGAAA	TTGCATAAAACTCCTCCCGCTA	136	57
*circ_0006661*	ACAGGAGCCGATATTTAGAGACC	TGCTGCCATAGCGACTTGCC	173	58
*circ_0020271*	TAATCCGCTGAAGATCCCT	CTCATCACACTGTACCCAT	205	54
*circ_0018320*	CTCTAGAAGCATTGGAGCAC	GCAGATGAAGAAAATACGCTCA	222	55
*β-actin*	TCCGTGACATCAAGGAGAAGC	CCGTGTTGGCGTAGAGGT	267	52–65

### Validation of the RNA-seq datas using RT-qPCR analysis

To verify the authenticity of RNA-seq datas. According to the manufacturer's protocols, five DE circRNAs that were the same as those analyzed using RT-PCR were subjected to real-time quantitative PCR (RT-qPCR) using 2 × the TransStart® Tip Green qPCR SuperMix (TransGen Biotech, Beijing, China) on an applied Roche LightCycler 480 (Roche Applied Science) platform. The reaction system and conditions of RT-qPCR were described by our previous article [38], the RNA samples and primers used for the RT-qPCR were the same as those used for the RT-PCR. The gene *ACTB* (encodes beta-actin) was used as housekeeping gene for standardization and the relative expression data were calculated with the 2^−∆∆Ct^ method [40].

### Functions enrichment analysis and interaction network construction

Gene ontology (GO) functions and Kyoto encyclopedia of genes and genomes (KEGG) enrichment pathways were analyzed to explore the important function of the host genes of DE circRNAs using GOseq R package and KOBAS-i software, respectively. *P*-value was < 0.05 was used as a threshold for the significantly enriched. For miRNA sequencing, total RNA was isolated from the above same liver tissue, six small RNA libraries were generated using NEBNext® Multiplex small RNA library Prep Set for Illumina® (NEB, USA). After quality control, libraries were sequenced on an Illumina Hiseq 2500 platform and 50 bp single-end reads were obtained. Clean data were obtained by removing reads containing adapters and low-quality. Then, the clean data were used to perform all the downstream analyses. In the present study, the differentially expressed miRNAs for (*P* ≤ 0.05 and |Log2 FC|≥ 1) were identified using the DEGseq R package. The target microRNAs of circRNAs were identified by miRanda (v.3.3a), the differentially expressed genes, circRNAs and their target microRNAs were used to build an interaction network based on our previous mRNA data (NCBI SRA accessions for mRNA sequencing data: SRR9291141, SRR9301148, SRR9301110, SRR9302163, SRR9302192, and SRR9302375) [38] and the unpublished miRNA data from the same liver samples. The circRNA-miRNA-gene network was visualized by using Cytoscape (v3.6.1) software.

### Identification and genotyping of target gene SNPs

DNA isolation for 1120 individuals were extracted from was performed using an EasyPure Blood Genomic DNA Kit (TransGen Biotech) according to the manufacturer's instructions. The PCR primers of the target genes designed in Table 4 using the Oligo7.0 software, and the specific PCR fragments were amplified using 20 mixed samples selected randomly from the above Hu sheep DNA samples, subsequently, the PCR products were sequenced to identify potential SNPs loci in the target genes. Finally, the identification of polymorphic loci in the target genes was carried out using KASPar assays according to a previous study [41]. The appropriate primers for the genotyping are listed in Table 4.

**Table 4:** The primers used for SNP identification and KASPar detection

primer's purpose	primer name	Primer sequence (5'–3')
Polymorphism	RTP4-F	CCCAGGCAGCTTGACTTCCA
	RTP4-R	GGTCAACACAGGACGCACAA
KAspar	RTP4_AlleleX	GAAGGTGACCAAGTTCATGCTCTGACCTTGATGTGGCCCTTG
	RTP4_AlleleY	GAAGGTCGGAGTCAACGGATTTCTGACCTTGATGTGGCCCTTA
	RTP4_Common	CCACAGCCCACCAGAGGGAC

### Data analyses

The results of the experimental data were statistically analyzed by using SPSS 21.0 for Windows Software. The differences of expression level were analyzed using a t-test for two groups. The association between genotypes and trait were analyzed by a linear model with the fixed effects: Y_ijk_ = μ + G_i_ + F_j_ + B_k_ + ε_ijk_, where Y_ijk_ is the phenotypic observation value, μ is the overall population mean, G_i_ is the effect of genotype, F_j_ is the effect of the j^th^ farm (j = 1, 2…6), B_k_ is the batch effect (k = 1, 2…5), ε is the random error. The data were indicated as means ± SD (standard deviation). A *P*-value < 0.05 was known as the statistically significant criterion. The Prism software (v5.0) was used to draw the bar graphs.

## Supplementary Information

**Additional file 1.**

## Data Availability

The sequencing reads of each sample in the present study have been deposited at GenBank with the Bioproject accession number PRJNA738639.

## Abbreviations

CircRNA: Circular RNA
CeRNAs: Competing endogenous RNAs
FI: Feed intake
IBW: Initial body weight
FBW: Final body weight
ADFI: Average daily feed intake
ADG: Average daily gain
MBW: Mid-test metabolic body weight
FCR: Feed conversion ratio
RFI: Residual feed intake
GO: Gene ontology
KEGG: Kyoto encyclopedia of genes and genomes
SNP: Single nucleotide polymorphism
