# Supplementary figures and images for: Identification and characterization of circular RNAs in association with the feed efficiency in Hu lambs
Source: BMC Genomics. 2022 Apr 10;23:288. doi: 10.1186/s12864-022-08517-5 (PMC8996647; doi:10.1186/s12864-022-08517-5)

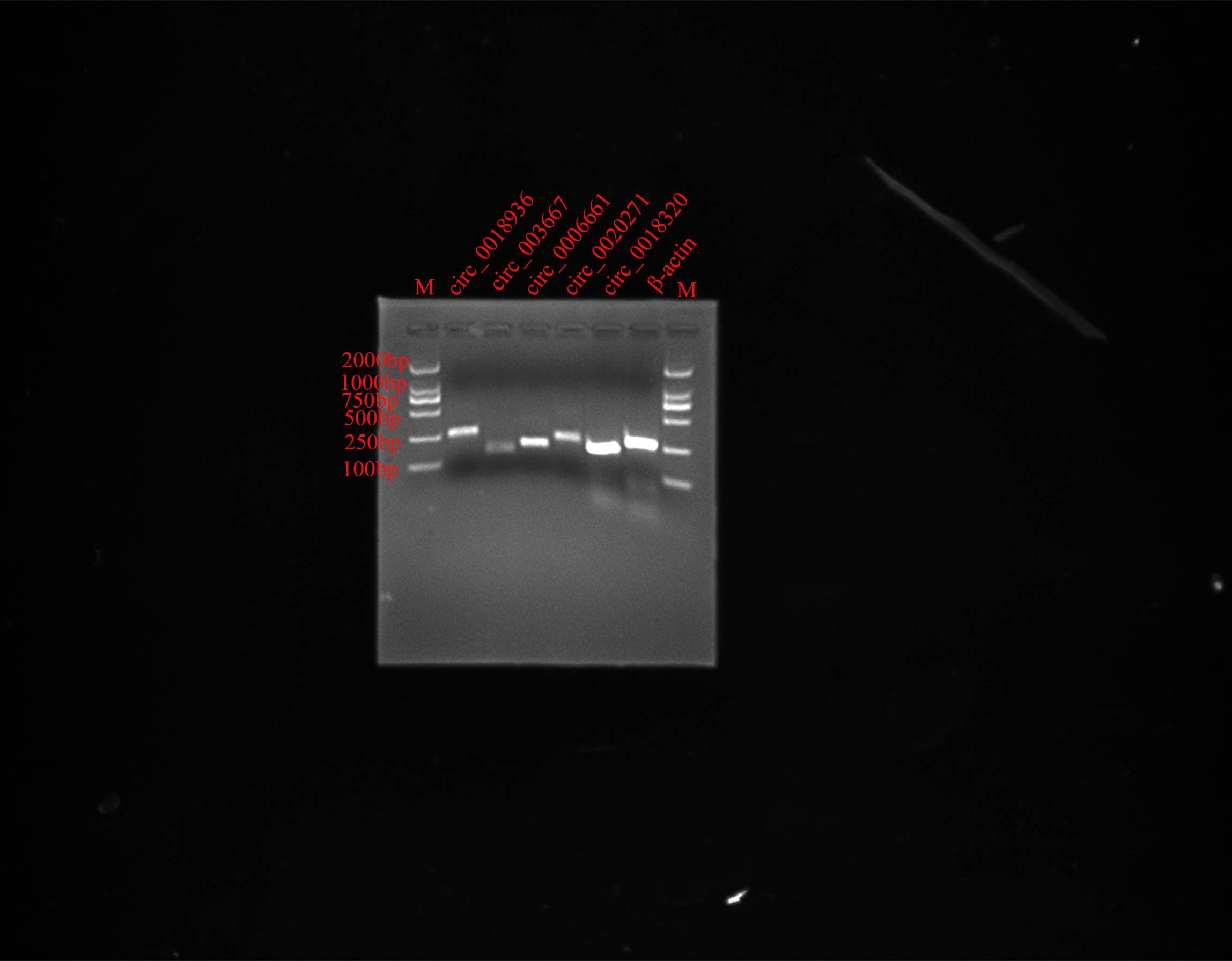

Supplement: Supplementary file 1 — Additional file 1. [file 12864_2022_8517_MOESM1_ESM.zip › Figure S1.tif]

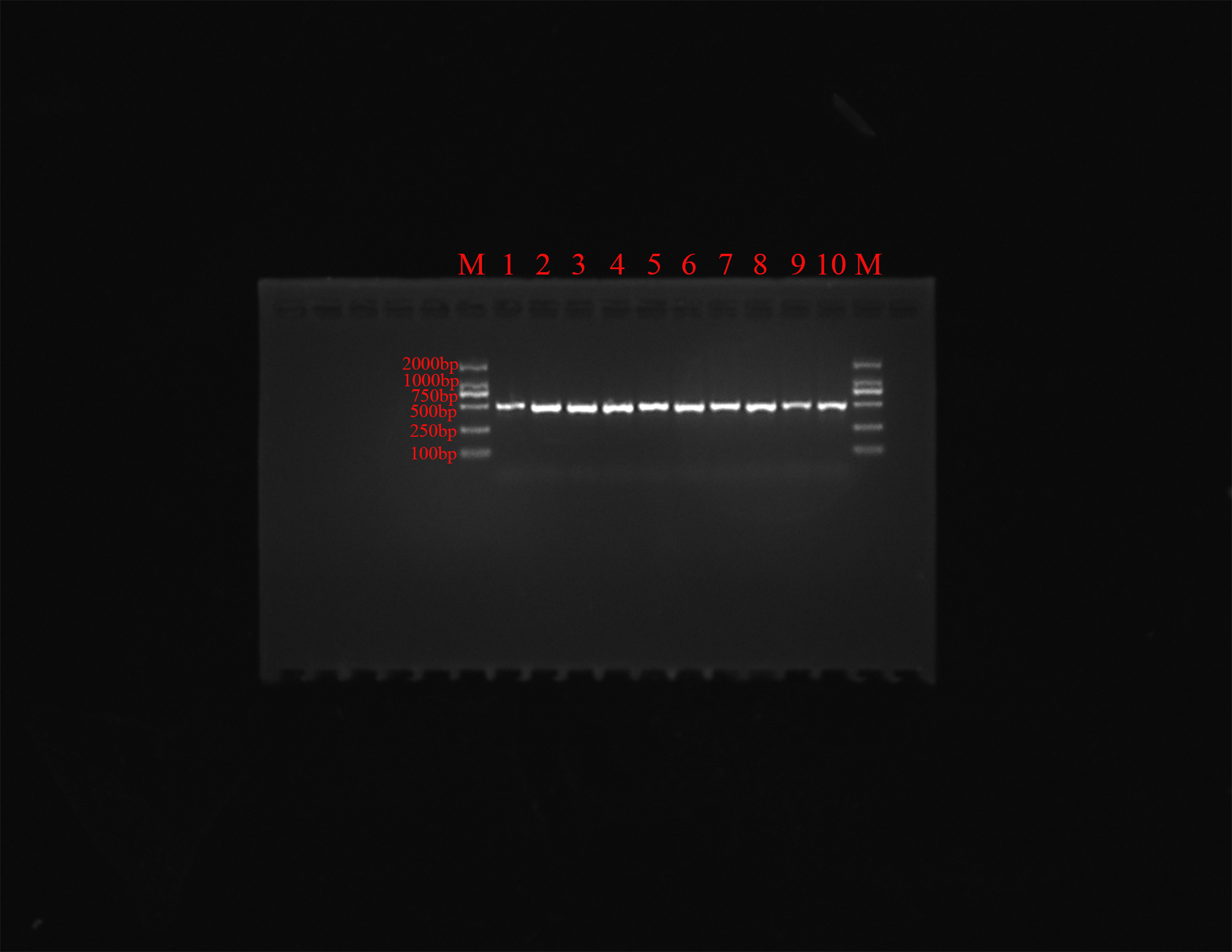

Supplement: Supplementary file 1 — Additional file 1. [file 12864_2022_8517_MOESM1_ESM.zip › Figure S2.tif]
